# Association Between Sleep Quality and Cognitive Complaints Among Young Adults With Migraine

**DOI:** 10.7759/cureus.83649

**Published:** 2025-05-07

**Authors:** Obasikene Johnson Ikechukwu, Marium Nadeem Khan, Saad Masood Khan, Muhammad Subhan, Muhammad Usairam Cheema, Nargis Abro, Oyelude Victor, Sana Khalid, Muhammad Soman, Lyba Zainab, Hamza Alvi

**Affiliations:** 1 Acute and General Medicine, Madonna University, Elele, NGA; 2 Internal Medicine, Shifa College of Medicine, Islamabad, PAK; 3 Internal Medicine, Government Nawaz Sharif Social Security Hospital, Lahore, PAK; 4 Internal Medicine, Jinnah Hospital, Allama Iqbal Medical College, Lahore, PAK; 5 General Medicine, Services Institute of Medical Sciences, Lahore, PAK; 6 Internal Medicine, People’s University of Medical and Health Sciences for Women, Nawabshah, PAK; 7 Family Medicine, MedCare Clinics, Niagara, CAN; 8 Medicine, NeuroWave Research Center, Islamabad, PAK; 9 Pediatrics, Combined Military Hospital (CMH) Kharian Medical College, Kharian, PAK; 10 Medicine, Heavy Industries Taxila Education City (HITEC) Institute of Medical Sciences, Taxila, PAK; 11 Medicine, York and Scarborough Teaching Hospitals National Health Service (NHS) Foundation Trust, York, GBR

**Keywords:** caffeine, cognitive complaints, migraine, sleep quality, young adults

## Abstract

Aim

Migraine is widely recognized as one of the most disabling neurological conditions, often accompanied by recurrent headache attacks along with disruptions in sleep and cognitive function. While previous studies have assessed sleep deficits and mental impairments in individuals with migraine, limited attention has been given to the intricate relationship between these variables, particularly in young adults. This study explores the association between sleep quality and cognitive complaints in individuals experiencing migraine, alongside an evaluation of migraine severity and caffeine consumption.

Methods

This cross-sectional study took place between December 2024 and March 2025 in Pakistan. A total of 300 participants aged 18 to 35 years with a prior clinical diagnosis of migraine were included. Participants completed a questionnaire containing scales that measured the Pittsburgh Sleep Quality Index (PSQI) alongside Migraine Disability Assessment (MIDAS) and Cognitive Failures Questionnaire (CFQ). Statistical assessments through Pearson’s correlation and ANOVA together with chi-square and linear regression testing were conducted with IBM SPSS Statistics for Windows, Version 26.0 (IBM Corp., Armonk, NY).

Results

The study indicated that migraine symptoms occurred in 226 (75%) of participants who also demonstrated sleep disorders at a rate of 167 (56%). The research established a strong positive connection between insufficient sleep quality and mental failures (r = 0.465, p < 0.01). Individuals who were between 27 and 30 years old and suffered from intense migraine symptoms experienced the worst sleep quality. People who consumed caffeine only occasionally reported the poorest sleep results (M = 120.1, SD = 24.9) along with the maximum cognitive failure outcomes (M = 77.8, SD = 19.7). The result of regression analysis showed sleep quality functioning as a crucial factor that predicts cognitive failure (β = 0.46, p < 0.01). The use of caffeine and the length of migraine episodes both demonstrated statistically significant links to both intense pain levels and sleep disruption impacts.

Conclusion

This research shows that migraine patients between 18 and 35 years old develop more cognitive issues because of their inadequate sleep patterns. Additional factors like occasional caffeine use along with stronger migraine pain levels combine to worsen sleep problems which call for specific therapeutic measures that combine behavior-based sleep strategies and limited caffeine intake. Future research based on objective sleep assessment needs to study this connection over time to validate these findings for clinical migraine care improvements.

## Introduction

Ranked by the World Health Organization (WHO) as the second most functionally impairing neurological disorder worldwide, migraine is characterized by episodes of moderate to severe and reversible headache sometimes preceded by sensory and systemic symptoms, including nausea, photophobia, and focal neurological deficits such as speech and motor disturbances [1]. Notorious for being highly debilitating and having considerable prevalence rates of about 17.1% to 19.2% in women and 5.6% to 7.2% in men, migraine creates an overwhelming financial as well as social burden on the patients' lives by inducing high medical treatment costs and isolating the patients from their families [2,3]. The International Classification of Headache Disorders (ICHD)-3 classifies migraine into three major categories, including migraine with aura, migraine without aura, and chronic migraine, with visual aura being the most prevalent complaint in the subset of patients experiencing aura before the onset of headache [4].

Sleep is an essential physiological process characterized by the state of absence of stimulatory and motor activity in the brain and is critical for inducing effective wakefulness throughout the day [5]. Sleep disturbance, commonly defined by compromised sleep quality and duration, trouble initiating sleep, and diurnal variation of the sleep cycle with excessive episodes of daytime sleepiness, is a major contributor to neurocognitive degeneration in older adults by stimulating global cortical thinning in the brain's age-sensitive regions [6,7]. Poor sleep quality stands to be one of the most well-researched causative factors in migraine pathology. It is also associated with inducing chronicity of this neurological condition by triggering major attacks.

Sleep disturbances contributing to poor sleep quality include frequent wakefulness during nighttime sleep, obstructive sleep apnea (OSA), poor sleep efficiency, and episodes of frequent sleep interruption caused by external factors. Considering the relationship between the fragmentation of sleep and its contribution to cognitive deterioration and loss of mental acuity in a broad subset of the population, it has been frequently postulated that migraine induces some degree of cognitive impairment in its sufferers during the interictal as well as the ictal periods [8,9].

Cognition refers to an individual's ability to accurately perform highly skilled mental tasks such as language processing, learning, problem-solving, and executive functioning. The neuro-psychological evaluation of cognition also includes the individual's ability to understand social cues and appropriately function in the sociocultural context [10]. Neurodegenerative diseases like mild cognitive impairment (MCI), Alzheimer's disease (AD), and dementia result in brain dysfunction that causes learning capacity decline combined with information memory loss and diminished working memory span, thus reducing short-term and long-term focus capabilities [11].

An extensive review of the literature regarding migraine's effect on the patient's cognition shows discordant results, with some studies suggesting that the migraine-related cognitive dysfunction domain includes complaints in information processing, memory retention, and difficulty in concentrating when performing important tasks [12,13]. Others suggest that despite migraine-inducing symptoms of impaired cognition in its sufferers, the long-term effect of repetitive attacks of this chronic neurological condition on permanent cognitive impairment is not clear [14]. Despite the contrasting evidence, the connection between sleep disturbances and cognitive impairment cannot be denied, which leads us to think about the effect of migraine-induced sleeplessness on cognitive impairment in individuals who have already been suffering from this condition for a long period.

Although migraine-related cognitive deficits and poor sleep quality are widely reported, the relationship between these two factors in young adults who already have heightened anxieties regarding career and family responsibilities remains insufficiently explored. This study aims to assess the correlation between sleep quality and cognitive complaints among adults with migraine, considering their daily functioning, quality of life, and potential for intervention. The objectives of this research are to evaluate the link between cognitive complaints and sleep quality among young adults diagnosed with migraine while also identifying the impact of migraine severity on cognitive complaints and sleep quality in this population. This paper also aims to explore whether poor sleep quality exacerbates cognitive complaints in young adults with migraine. By studying this subject matter, we aim to fill an important research gap regarding the effect of migraine on cognitive impairment in adults already suffering from poor sleep quality due to multiple reasons. Understanding this critical relationship between migraine-induced cognitive disturbance can help in the development of targeted therapeutic models, especially in the field of sleep and behavioral medicine.

Objective

This study aimed to investigate the relationship between sleep quality and cognitive complaints among young adults diagnosed with migraine. By examining patterns across variables such as migraine severity, duration, and caffeine consumption, the research sought to determine whether poor sleep quality significantly contributes to cognitive difficulties in this population. The study further explored how behavioral factors and migraine-related pain intensity influence mental performance and daily functioning, with the goal of identifying potential targets for therapeutic intervention.

## Materials and methods

Study design

This quantitative cross-sectional study was carried out in Pakistan from December 2024 to March 2025 on patients diagnosed with migraines according to the International Headache Society (IHS) criteria [4]. Informed consent was taken from the participants, and complete anonymity was maintained. Ethical clearance was provided by the Institutional Review Board of NeuroWave Research Institute, Islamabad, which approved the study design (IRB-2025-0067).

Sample size and selection criteria

The sample size was calculated using the formula for an infinite population, as the number of young adults with migraine in the target population was unknown. The formula used is:

Sample size = (Z² × p × (1 - p)) / d²

In this formula, Z represents the Z-value for a 95% confidence level (1.96), p was set at 0.5 due to the absence of prior data on expected prevalence, and d denotes the margin of error set at 0.05. Substituting these values into the formula yielded a sample size of 385. However, 300 participants were included in the study due to time constraints and resource availability. This sample size was considered sufficient to detect meaningful associations while maintaining statistical validity, as supported by similar cross-sectional studies in the literature [15].

Strict selection criteria were designed to include participants. The criteria for inclusion were as follows: (1) Participants aged 18-35 years; (2) Participants having chronic or episodic migraine, according to the International Headache Society (IHS) criteria [4]; (3) Participants have no neurological disorders other than migraine; (4) Participants having no active psychiatric conditions, including depression or anxiety; (5) Participants having the capacity to provide consent and take part in the study.

Participants were excluded if they had a history of neurological disorders such as epilepsy and stroke, or were diagnosed with any psychiatric condition, such as schizophrenia and bipolar disorder. Pregnant or breastfeeding individuals were excluded, along with participants currently using medications that affect sleep or cognitive functions, such as sedatives or antidepressants. Additionally, participants not falling within the inclusion criteria were excluded as well.

Data collection

A non-probability convenience sampling technique was used for data collection. A specific questionnaire was designed and consisted of four parts: (1) Demographics which consisted of age, gender, educational status, and characteristics of migraine and sleep; (2) the Pittsburgh Sleep Quality Index (PSQI) (Appendix A) developed by Buysse et al. (1989), was used to assess sleep components: sleep quality, sleep duration, sleep latency, habitual sleep efficiency, daytime dysfunction, use of sleeping medication and sleep disturbances (a global score below 5 meant good sleep quality and a global score above 5 showed poor sleep quality) [16]; (3) the Migraine Disability Assessment Test (MIDAS) (Appendix B) developed by Stewart et al. (2001), was used to check the impact of migraine on daily life and was characterized by four grades with grade 1 (0-5) showing little or no disability, grade 2 (6-10) showing mild disability, grade 3 (11-20) showing moderate disability and grade 4 (21+) showing severe disability [17]; (4) the Cognitive Failures Questionnaire (CFQ) (Appendix C) developed by Broadbent et al. (1982), was used to assess cognitive failures experienced by the participants and consisted of three domains including forgetfulness, distractibility, and false triggering whereby greater frequency of cognitive failures experienced by the patients was indicated by higher scores [18]. The survey was conducted physically through in-person administration at various hospital and clinical outpatient settings across different cities in Pakistan.

Statistical analysis

The data analysis was performed using IBM SPSS Statistics, Version 26 (IBM Corp., Armonk, NY). This statistical software was used to calculate frequencies alongside percentages which described the demographics and clinical characteristics variables. The examination of the connection between sleep quality and cognitive failures required a Pearson correlation test. ANOVA one-way showed whether the mean variations between different groups created significant differences while measuring PSQI and CFQ. The investigation of sleep quality influence on cognitive failures used a linear regression model with a 95% confidence interval. The Chi-square test analyzed the relationships between various demographic characteristics along with pain levels and patterns observed during caffeine consumption. The results showed statistical significance at a p-value lower than 0.05 throughout this research.

## Results

Table 1 demonstrates the demographic and clinical information for 300 participants (N = 300). Among these, 226 participants (75%) reported experiencing migraine headaches. All migraine-specific variables (duration, frequency, severity, and medication use) are based solely on this subgroup (n = 226). The majority were aged between 21 and 23 years (52%), and male participants constituted a larger proportion (76%). Most participants were undergraduates (45%) and employed either full-time (41%) or part-time (30%). Sleep-related findings showed that 56% had sleep disorders, and 39% slept for four to six hours daily. Regular caffeine use was reported by 52%, and 83% used sleep aids.

**Table 1:** Demographic characteristics of participants (N=300) f: frequency; %: percentage

Variable	f	%
Age	-	-
18-20 years	86	29
21-23 years	156	52
24-26 years	42	14
27-30 Years	16	5
Gender	-	-
Male	229	76
Female	71	24
Educational Level	-	-
High school or below	58	19
Undergraduate	135	45
Postgraduate	90	30
Doctorate	17	6
Employment Status	-	-
Student	63	21
Employed (full-time)	122	41
Employed (part-time)	89	30
Unemployed	26	9
Migraine headache	-	-
Yes	226	75
No	74	25
Duration of migraine	-	-
Less than 6 months	99	33
6 months – 1 year	135	45
1-2 years	45	15
More than 2 years	21	7
Frequency of migraine	-	-
Rarely	62	21
Occasionally	125	42
Frequently	77	26
Daily	36	12
Severity of pain	-	-
Mild	77	26
Moderate	124	41
Severe	68	23
Very severe	31	10
Migraine medication	-	-
Yes	212	71
No	88	29
Sleep disorder	-	-
Yes	167	56
No	84	28
Not sure	49	16
Sleep hours	-	-
Less than 4 hours	99	33
4-6 hours	116	39
6-8 hours	67	22
More than 8 hours	18	6
Regular use of caffeine	-	-
Yes	156	52
No	78	26
Occasionally	66	22
Use of sleep aids	-	-
Yes	249	83
No	51	17

Table 2 shows intercorrelations between the key study variables. The PSQI had a moderate positive correlation with the CFQ (r = 0.409, p < 0.01), indicating that poor sleep quality is significantly associated with increased cognitive complaints. A weak but statistically significant positive correlation was observed between PSQI and MIDAS (r = 0.046, p < 0.01). Interestingly, CFQ and MIDAS scores demonstrated a negative correlation (r = -0.007, p < 0.01), though statistically significant, it was practically negligible. These findings suggest that poor sleep quality may have a stronger impact on cognitive difficulties than on migraine-related disability in this population.

**Table 2:** Intercorrelations between study variable * p<0.01 considered statistically significant

Variable	1	2	3
Pittsburgh Sleep Quality Index	-	-	-
Cognitive Failure Questionnaire	0.409^*^	-	-
Migraine Disability Assessment Test	0.046^*^	-.007^*^	-

Table 3 compares variables by age group. Participants aged 27-30 years (N=16, 5%) reported the worst sleep quality (M=136.3) with significant differences across age groups (F(3,296) = 22.74, p < 0.01, η² = 0.187). MIDAS scores also significantly increased with age (F(3,296) = 23.79, η² = 0.381), indicating greater migraine disability among older participants. However, CFQ scores did not significantly differ across groups (F = 0.446, η² = 0.005), suggesting that cognitive failures were not age-dependent in this sample.

**Table 3:** Comparison of variables (age) M: mean; SD: standard deviation; F: F-ratio; η2: effect size * p<0.01 considered statistically significant

Variable	18-20 years	21-23 years	24-26 years	27-30 years	F (3,296)	η2
	M±SD	M±SD	M±SD	M±SD		
Pittsburgh Sleep Quality Index	95.8±20.8	105.4±20.9	118.9±25.0	136.3±15.3	22.74^*^	0.187
Cognitive Failure Questionnaire	73.1±12.8	74.0±11.6	74.3±17.2	77.5±28.3	0.446^*^	0.005
Migraine Disability Assessment Test	27.9±9.8	34.3±10.1	39.8±12.1	47.9±11.0	23.79^*^	0.381

Table 4 presents differences based on migraine duration. Sleep quality scores did not significantly vary with duration (F = 0.216, η² = 0.002). Participants with migraines for more than two years (N=21, 7%) had slightly worse CFQ and MIDAS scores but not significantly. The minimal effect sizes across sleep quality, cognitive failure, and migraine disability (η² < 0.04) suggest that migraine chronicity had limited influence on these outcomes in this sample.

**Table 4:** Comparison of variables (duration of migraine) M: mean; SD: standard deviation; F: F-ratio; η2: effect size * p<0.01 considered statistically significant

Variable	Less than 6 months	6 months-1 year	1-2 years	More than 2 years	F (3,296)	η2
	M±SD	M±SD	M±SD	M±SD		
Pittsburgh Sleep Quality Index	105.2±23.8	107.1±23.9	104.8±18.1	108.1±29.5	0.216^*^	0.002
Cognitive Failure Questionnaire	72.2±16.2	74.7±12.2	73.6±7.18	79.2±22.7	1.654^*^	0.017
Migraine Disability Assessment Test	34.9±12.6	33.9±10.8	31.2±8.5	36.4±15.3	1.426^*^	0.036

Table 5 examines variables by migraine pain severity. Participants with very severe pain (N=31, 10%) had the worst sleep scores (M=116.9) with a statistically significant difference (F = 4.507, p < 0.01, η² = 0.044). MIDAS scores also rose with increasing pain intensity (F = 2.513, p < 0.05, η² = 0.061). However, CFQ scores remained statistically unchanged across severity groups (F = 0.427, η² = 0.004), showing that pain severity affects sleep and disability more than cognitive complaints.

**Table 5:** Comparison of variables (pain severity) M: mean; SD: standard deviation; F: F-ratio; η2: effect size * p<0.01 considered statistically significant

Variable	Mild	Moderate	Severe	Very severe	F (3,296)	η2
	M±SD	M±SD	M±SD	M±SD		
Pittsburgh Sleep Quality Index	99.5±19.4	106.9±24.5	107.5±21.1	116.9±28.7	4.507^*^	0.044
Cognitive Failure Questionnaire	72.2±11.9	74.3±14.9	74.2±10.2	77.0±21.1	0.427^*^	0.004
Migraine Disability Assessment Test	31.9±10.3	33.7±12.1	35.0±11.9	38.3±9.9	2.513^*^	0.061

Table 6 explores the impact of sleep disorders. Participants not sure about having a sleep disorder (N=49, 16%) had the worst sleep quality and highest MIDAS scores (F = 15.391 & 14.636, respectively). Sleep disorder status did not significantly affect CFQ scores (F = 0.982, η² = 0.007). These results indicate that perceived uncertainty about sleep health correlates with increased migraine disability and poor sleep.

**Table 6:** Comparison of variables (sleep disorders) M: mean; SD: standard deviation; F: F-ratio; η2: effect size * p<0.01 considered statistically significant

Variable	Yes	No	Not sure	F (2,297)	η2
	M±SD	M±SD	M±SD		
Pittsburgh Sleep Quality Index	101.3±21.2	107.2±24.8	121.4±22.2	15.391^*^	0.094
Cognitive Failure Questionnaire	73.1±13.1	74.6±10.9	76.1±20.6	0.982^*^	0.007
Migraine Disability Assessment Test	31.7±10.3	34.4±11.8	41.3±12.1	14.636^*^	0.252

Table 7 analyzes caffeine use patterns. Participants who used caffeine occasionally (N=66, 22%) showed the worst sleep quality (M=120.1) and highest CFQ (M=77.8) and MIDAS scores (M=38.9). These differences were all statistically significant (F = 21.234, 3.672, 12.373, respectively), with moderate to strong effect sizes. Erratic caffeine use appears strongly associated with sleep dysfunction, cognitive issues, and increased migraine impact.

**Table 7:** Comparison of variables (caffeine use) M: mean; SD: standard deviation; F: F-ratio; η2: effect size * p<0.01 considered statistically significant

Variable	Yes	No	Occasionally	F (2,297)	η2
	M±SD	M±SD	M±SD		
Pittsburgh Sleep Quality Index	99.2±19.6	108.4±23.9	120.1±24.9	21.234^*^	0.125
Cognitive Failure Questionnaire	73.5±9.0	71.6±16.1	77.8±19.7	3.672^*^	0.024
Migraine Disability Assessment Test	31.2±10.2	35.6±13.2	38.9±10.4	12.373^*^	0.222

Linear regression results in Table 8 show that neither PSQI nor MIDAS scores were significant predictors of CFQ when both variables were entered simultaneously. The model showed small, non-significant coefficients (PSQI B = 0.035, β = 0.058; MIDAS B = -0.037, β = -0.030). This suggests that while these factors correlate individually with cognitive complaints, they do not independently predict them in a combined model.

**Table 8:** Linear regression for study variables B: coefficient; SE: standard error; β: standardized coefficient; LL: lower limit; UL: upper limit; Cl: confidence interval * p<0.01 considered statistically significant

Variable	B	95% Cl		SE	β
		LL	UL		
Constant	71.537	63.8	79.2	3.91	-
Pittsburgh Sleep Quality Index	0.035	-.040	.110	0.04	0.058^*^
Migraine Disability Assessment Test	-0.037	-.190	.116	0.08	-0.030^*^

Table 9 provides a cross-tabulation of pain severity and caffeine use by migraine duration and sleep disorder status. Among those with less than six months of migraine (N=99, 33%), moderate pain was most common. Longer migraine durations showed increased very severe pain (p = 0.006, χ² = 22.9). Caffeine use also varied significantly with migraine duration (p = 0.04, χ² = 13.2). No significant relationship was found between sleep disorder status and pain severity (p = 0.71), but a strong association existed between sleep disorder and caffeine use (p < 0.001, χ² = 32.58). These patterns highlight how chronic migraine and sleep health influence caffeine behaviors and pain perceptions.

**Table 9:** Descriptive statistics of demographic variables f: frequency; %: percentage; p: level of significance; p-values calculated using the chi-square test; the significance level is set at p < 0.05

Variables	f	Mild	Moderate	Severe	Very severe	p	x^2^	Yes	No	Occasionally	p	x^2^
Duration of migraine	-	-	-	-	-	-	-	-	-	-	-	-
Less than 6 months	99	23	50	20	6	.006	22.9	49	33	17	.04	13.2
6 months-1 year	135	30	54	34	17	-	-	79	23	33	-	-
1-2 years	45	20	14	9	2	-	-	21	15	9	-	-
More than 2 years	21	4	6	5	6	-	-	7	7	7	-	-
Sleep disorders	-	-	-	-	-	-	-	-	-	-	-	-
Yes	167	47	66	38	16	.71	3.76	103	38	26	.000	32.58
No	84	21	34	21	8	-	-	42	26	16	-	-
Not sure	49	9	24	9	7	-	-	11	14	24	-	-

## Discussion

According to our intercorrelation and linear regression analysis data, our research investigation demonstrates that sleep quality plays a significant role in cognitive difficulties among young adults experiencing migraine. Sleep quality was poorest among participants in the higher age group (20-27 years), with severe migraine pain, occasional caffeine use, and in those who were unsure about having a sleep disorder. Our analysis indicated that participants with no caffeine intake had better cognitive outcomes than those with occasional caffeine intake. There was a significant association indicating that young adults with an increased duration of migraine use more caffeine and experience greater severity of pain. Those with greater caffeine use had more sleep-related issues.

A similar outcome outlining the association between poor sleep quality and greater cognitive difficulties in migraineurs is observed in a study conducted in Korea, where the average sleep duration of migraineurs with subjective cognitive decline (SCD) was six hours, significantly less than the recommended seven to eight hours [19]. A double-blinded study by Esmael et al. reported a higher PSQI score, decreased sleep efficiency, sleep time, and rapid eye movement (REM) percentage in migraineurs with subjective cognitive impairment (SCI) than in those with no SCI [20].

Migraineurs who slept less than six hours a day have shown severe headache patterns and low sleep durations, as reported by Lee et al. and Esmael et al. [19,20]. Patients with chronic migraine exhibit a phenomenon known as pain catastrophizing, which is a negative cognitive and emotional response that increases pain symptoms, helplessness, and pessimism. This phenomenon is a significant predictor of sleep quality [21]. Thus, less duration of sleep and pain catastrophizing may be responsible for a decline in cognitive function and, at the same time, reinforce our outcome by associating the poorest sleep quality with severe migraine pain [19-21]. On the contrary, a study on healthy young adults revealed no association between sleep quality and cognitive function. This may be due to peak cognitive performance in young adults and the absence of clinical disorders [22].

The outcomes of our study established that migraineurs with greater caffeine use had more sleep-related issues. Previous studies have reinforced this relationship between caffeine intake and sleep quality [23-25]. The poorest sleep quality was observed in occasional caffeine users compared to those who consumed it regularly, which can be attributed to the fact that regular caffeine use over several days may increase adenosine receptors in the brain, leading to tolerance to the acute effect of caffeine [26]. Stimulating and prolonged alerting effects of caffeine may have a role in sleep disorders and, therefore, trigger migraine attacks and increase their duration, similar to what our results depict [27]. However, a study by Mittleman et al. suggests no correlation between habitual caffeine intake and headache frequency and duration, indicating that drinking more caffeinated beverages than a person typically does would be harmful [28].

The highest cognitive decline was noted in occasional caffeine users, rather than regular users, in our study, which might be the result of some participants going through caffeine withdrawal. Contrary to the literature available [29,30], caffeine users showed lower cognitive failure scores than occasional users. This may be attributed to pre-existing variations influencing the results, as these groups are self-selected [31]. Whereas some studies establish a strong association between cognitive impairment with a duration of migraine and pain severity [32,33], the results of our study show no such relation. The outcome of a previous meta-analysis conducted on cognitive impairment in migraine reveals that cognitive dysfunction may be a result of central nervous system dysfunction rather than the duration and severity of headache [34].

This study had some limitations. The study design, as a cross-sectional type, failed to establish causal relationships. Future research with extended duration should analyze the link between migraine and sleep quality, together with cognitive impairment, to obtain a better understanding. Since this study's sample is small, a larger sample would provide more substantial outcomes. Moreover, we used a survey questionnaire to inquire about migraine and related effects, automatically producing unavoidable reporting biases. The PSQI questionnaire was used to assess sleep quality, which might be subjective. Instead, polysomnography should be used.

## Conclusions

The research investigation shows how migraine patients facing poor sleep quality develop elevated cognitive difficulties during their young adult years. The research facts indicate that occasional use of caffeine combined with severe migraine headache intensity together with an unclear awareness of sleep disorders leads to decreased sleep quality and poor cognitive outcomes. Study participants who abstained from caffeine usage showed superior cognitive abilities to those who occasionally consumed caffeine cylinders. This could be due to caffeine withdrawal effects and natural differences among participants. Literature findings neither fully nor partly confirm these results because of methodological discrepancies and sample characteristics. Understanding migraine, sleep, and cognitive relationships requires additional studies combining longitudinal methods and objective monitoring using polysomnography to validate previously reported findings.

## Appendices

Appendix A 

**Table 10:** Pittsburgh Sleep Quality Index Developed by Buysse et al. (1989) [16]

Items	Responses
During the past month, what time have you usually gone to bed at night?	-
During the past month, how long (in minutes) has it usually taken you to fall asleep each night?	-
During the past month, what time have you usually gotten up in the morning?	-
During the past month, how many hours of actual sleep did you get at night? (This may be different than the number of hours you spent in bed.	-
During the past month, how often have you had trouble sleeping because you	-
Cannot get to sleep within 30 minutes	-
Wake up in the middle of the night or early morning	-
Have to get up to use the bathroom	-
Cannot breathe comfortably	-
Cough or snore loudly	-
Feel too cold	-
Feel too hot	-
Had bad dreams	-
Have pain	-
Other reason(s), please describe	-
How often during the past month have you had trouble sleeping because of this?	-
During the past month, how would you rate your sleep quality overall?	-
During the past month, how often have you taken medicine to help you sleep (prescribed or “over the counter”)?	-
During the past month, how often have you had trouble staying awake while driving, eating meals, or engaging in social activity?	-
During the past month, how much of a problem has it been for you to keep up enough enthusiasm to get things done?	-
Do you have a bed partner or roommate?	-
If you have a roommate or bed partner, ask him/her how often in the past month you have had	-
Loud snoring	-
Long pauses between breaths while asleep.	-
Legs twitching or jerking while you sleep	-
Episodes of disorientation or contusion during sleep	-
Other restlessness while you sleep, please describe	-

Appendix B 

**Table 11:** Migraine Disability Assessment Test Developed by Stewart et al. (1989) [17]

Items	Response
On how many days in the last 3 months did you miss work or school because of your headaches?	-
How many days in the last 3 months was your productivity at work or school reduced by half or more because of your headaches? (Do not include days you counted in question 1 where you missed work or school.)	-
On how many days in the last 3 months did you not do household work (such as housework, home repairs and maintenance, shopping, caring for children and relatives) because of your headaches?	-
How many days in the last 3 months was your productivity in household work reduced by hall of more because of your headaches? (Do not include days you counted in question 3 where you did not do household work.)	-
On how many days in the last 3 months did you miss family, social or leisure activities because of your headaches?	-
What your Physician will need to know about your headache:	-
On how many days in the last 3 months did you have a headache? (If a headache lasted more than 1 day, count each day.)	-
On a scale of 0-10, on average how painful were these headaches? (where 0-no pain at all, and 10-pain as bad as it can be.)	-

Appendix C 

**Table 12:** Cognitive Failure Questionnaire Developed by Broadbent et al. (1982) [18]

Items	Responses
1.Do you read something and find you haven’t been thinking about it and must read it again?	
2. Do you find you forget why you went from one part of the house to the other?	
3. Do you fail to notice signposts on the road?	
4. Do you find you confuse right and left when giving directions?	
5. Do you bump into people?	
6. Do you find you forget whether you've turned off a light or a fire or locked the door?	
7. Do you fail to listen to people's names when you are meeting them?	
8. Do you say something and realize afterwards that it might be taken as insulting?	
9. Do you fail to hear people speaking to you when you are doing something else?	
10.Do you lose your temper and regret it?	
11.Do you leave important letters unanswered for days?	
12.Do you find you forget which way to turn on a road you know well but rarely use?	
13. Do you fail to see what you want in a supermarket (although it’s there)?	
14.Do you find yourself suddenly wondering whether you've used a word correctly?	
15. Do you have trouble making up your mind?	
16.Do you find you forget appointments?	
17. Do you forget where you put something like a newspaper or a book?	
18. Do you find you accidentally throw away the thing you want and keep what you meant to throw away, as in the example of throwing away the matchbox and putting the used match in your pocket?	
19. Do you daydream when you ought to be listening to something?	
20. Do you find you forget people’s names?	
21. Do you start doing one thing at home and get distracted into doing something else (unintentionally)?	
22. Do you find you can't quite remember something, although it's "on the tip of your tongue"?	
23. Do you find you forget what you came to the shops to buy?	
24. Do you drop things?	
25. Do you find you can’t think of anything to say?	
